# The importance of monitoring cerebral oxygenation in non brain injured patients

**DOI:** 10.1007/s10877-023-01002-8

**Published:** 2023-04-12

**Authors:** Chiara Robba, Denise Battaglini, Francesco Rasulo, Francisco A. Lobo, Basil Matta

**Affiliations:** 1 Anesthesia and Intensive Care, IRCCS Policlinico San Martino, Genoa, Italy; 2 Department of Surgical Science and Integrated Diagnostics, University of Genoa, Genoa, Italy; 3 Department of Anesthesia and Intensive Care, Spedali Civili University Affiliated Hospital of Brescia, Brescia, Italy; 4 Institute of Anesthesiology, Cleveland Clinic, Abu Dhabi, United Arab Emirates; 5 Neurocritical Care Unit, Cambridge University Hospitals, Cambridge, UK

**Keywords:** Neuromonitoring, Operatory room, Intensive care unit, Cerebral oxygenation

## Abstract

Over the past few years, the use of non-invasive neuromonitoring in non-brain injured patients has increased, as a result of the recognition that many of these patients are at risk of brain injury in a wide number of clinical scenarios and therefore may benefit from its application which allows interventions to prevent injury and improve outcome. Among these, are post cardiac arrest syndrome, sepsis, liver failure, acute respiratory failure, and the perioperative settings where in the absence of a primary brain injury, certain groups of patients have high risk of neurological complications. While there are many neuromonitoring modalities utilized in brain injured patients, the majority of those are either invasive such as intracranial pressure monitoring, require special skill such as transcranial Doppler ultrasonography, or intermittent such as pupillometry and therefore unable to provide continuous monitoring. Cerebral oximetry using Near infrared Spectroscopy, is a simple non invasive continuous measure of cerebral oxygenation that has been shown to be useful in preventing cerebral hypoxemia both within the intensive care unit and the perioperative settings. At present, current recommendations for standard monitoring during anesthesia or in the general intensive care concentrate mainly on hemodynamic and respiratory monitoring without specific indications regarding the brain, and in particular, brain oximetry. The aim of this manuscript is to provide an up-to-date overview of the pathophysiology and applications of cerebral oxygenation in non brain injured patients as part of non-invasive multimodal neuromonitoring in the early identification and treatment of neurological complications in this population.

## Introduction

The application of non-invasive neuromonitoring tools in non primarily brain injured patients has increased over the last years [1, 2]. A number of clinical indications have been suggested for the use of different non invasive neuromonitoring tools. Among these, severe respiratory failure with or without extracorporeal membrane oxygenation (ECMO) [3, 4], trauma, cardiac arrest, liver failure, intra-arterial thrombolysis during endovascular treatment [5, 6], and sepsis are among the conditions where despite the absence of a primary cerebral damage, neurological complications are common and can affect patients’ outcome [1]. The benefits of non invasive methods include safety, availability, and the provision of repeatable continuous data at the bedside, therefore helping clinicians detecting deterioration in neurologic function and earlier intervention [7].

Among the different neuromonitoring methods [8–10], the use of cerebral oxygenation has been recently suggested [11]. Of course the use of cerebral oximetry is not new as it has been a mainstay of managing patient with traumatic brain injury for years, and there are three methods currently available: jugular bulb saturation, which allows an estimation of global oxygenation and require an invasive catheter to be positioned in the jugular bulb; brain tissue oxygenation, which is now considered the gold standard and measures focal oxygenation through a Clark electrode; Near-infrared spectroscopy (NIRS), which measures tissue oximetry non invasively providing an estimate of the balance between oxygen delivery and metabolic needs of the brain. NIRS, being the only non invasive method of estimating brain oxygenation today, it seems to be the most appropriate modality for use in patients who are undergoing surgery or sedation in the intensive care where the primary pathology is not brain injury. The aim of this review is to provide an up-to-date view on the main technical and pathophysiological characteristics of NIRS, as well as the most frequent clinical conditions which have the potential of benefiting from the application of this technology for the detection of neurological complications in non-brain injured patients, as well as to describe current limitations in its applicability and future directions.

### Cerebral oxygenation

Brain health depends on close matching of metabolic demands to appropriate delivery of oxygen and nutrients, and removal of cellular waste. The oxygen level in cerebral tissue is a crucial element that impacts nerve and glial cell functions. The weight of the brain is only 2% of the human body, but cerebral tissue uses approximately 25% of the glucose and 20% of the oxygen delivered to the entire body to function normally [12]. Cerebral oxygen delivery is determined by blood oxygen content (haemoglobin, saturation and small amount of dissolved oxygen) and cerebral blood flow, which is dependant in large parts on cardiac output (stroke volume x heart rate), and well as other factors such as carbon dioxide tension. In physiological conditions, total blood flow in the brain is constant because of cerebral pressure autoregulation which regulates vascular resistance in the large arteries, of vascular resistance, as well as parenchymal arterioles basal tone.

Oxygen consumption is 3.5 mL of oxygen/100 g tissue/1 min, of which 75–80% of the energy consumed by neurons to restore the neuronal membrane potentials is lost during depolarization. Diffusion of oxygen to the cerebral tissue is determined by the geometry of capillaries and the metabolism of tissue [12]. Extraction of oxygen is inversely proportional to blood flow at constant metabolism and directly proportional to metabolism at constant flow. A reduction in oxygen delivery increases oxygen extraction. When CBF is reduced by 50–60%, the consequent elevation of oxygen extraction is insufficient to maintain a constant cerebral metabolic rate of oxygen (CMRO2).

The oxygen cascade is a multistep physiologic pathway, where oxygen is transported from the atmosphere to mitochondria. This process requires the integration of different patterns and respiratory, cardiovascular, microcirculatory, and mitochondrial processes [12–14]. The brain, which possesses a high metabolic demand and commensurate vulnerability to interruptions in its oxygen supply, is reliant upon consistent perfusion and delivery of oxygen to maintain homeostasis. The regulation of cerebral blood flow (CBF) is crucial to be steady according to the current needs. Adequate CBF is delivered by four main mechanisms: cerebral vasculature response to changes in cerebral perfusion pressure (autoregulation), vascular reactivity to vasoactive stimuli, a response to local changes in neural activity on the cognitive stimuli [neurovascular coupling (NVC)] and endothelium-dependent responses. CBF is essential to support activity of neurons and other brain cells and any disruptions in CBF regulation at baseline, temporal, or regional level, can progress into neurodegenerative diseases.

### NIRS: what it is and what it is not

**Fig. 1:**
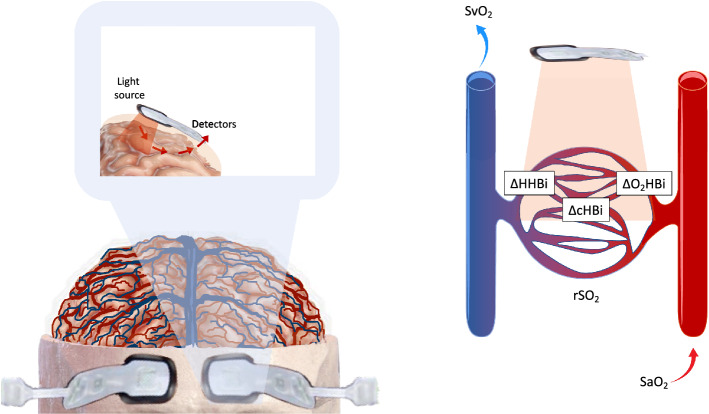
Near infrared Spectroscopy technology. *SaO2* systemic oxygen saturation, *rSO*_*2*_ cerebral oxygen saturation, *∆O2HBi* oxygenated component of hemoglobin, *∆HHBi* deoxygenated component of Hemoglobin, *∆cHBi* sum of oxygenated and deoxygenated hemoglobin

NIRS measures tissue oxygenation by capturing reflected near-infrared light passing through the cranial bone to the underlying cerebral tissue utilizing the transparency of the scalp and skull to infrared light and the differences in absorption spectra between oxyhemoglobin and deoxyhemoglobin to quantify the local oxygen saturation of hemoglobin in the brain. This method assumes that the sampled tissue comprises approximately 75% of venous 25% of arterial blood [15] (Fig. 1). NIRS uses a wavelength range between 600 and 1000 nm, and its mechanism is based on two main factors: scattering—which is the dominant effect in biological tissue and is related to microscopic refractive index changes inside the tissue; and absorption, --which is related to the loss of a photon caused by the presence of a particular chromophores inside the tissue that convert light intensity into other types of energy. Therefore, because each chromophore has a specific spectral shape, each one of them will contribute differently to the overall absorption. The multiwavelength light source used in NIRS utilizes in fact specific wavelengths able to separate the contribution of each chromophore and therefore to quantify its concentration [16–18].

The basic functioning of NIRS machines relies on a single point data acquisition [19], which consists in the use of a continuous light which is emitted into the tissue, and then the transmitted attenuated light is collected few centimetres away. In this way, only the changes in light attenuation, defined as the variation and reduction of the transmitted/reflected light intensity from the emitted light are collected, and these changes can calculate, according to the modified Beer–Lambert law the modifications in oxygenated [HbO_2_] and deoxygenated Hemoglobin [HHb] concentrations [20]. The tissue saturation reflects the ratio between the concentration of [HbO_2_] and the concentration of total haemoglobin ([HbT] = [HbO_2_] + [HHb]) [21] (Fig. 1). This has been widely exploited by commercial brain oximeters [22]. Recently, some extensions of this technique have been suggested, with the use of the spatially resolved spectroscopy (SRS) technique, based on the measurement of the light attenuation at several source/detector separations, and the ability to obtain absolute tissue oxygen saturation values (referred as tissue oxygenation index (TOI) or tissue saturation (StO_2_) in the literature) [23].

As for the pathophysiological factors above mentioned, NIRS is also a promising tool for portable, continuous, long-term and noninvasive monitoring of regional CBF at bedside. The difference between the tissue concentrations of oxy-hemoglobin and deoxy-hemoglobin ([Hb]), is a surrogate of CBF, and has been observed to be highly correlated with CBF [24]. However, this is not only sensitive to CBF changes, but also to cerebral metabolic rate of oxygen (CMRO_2_) and cerebral blood volume (CBV). A number of indices have been developed over recent years in an attempt to derive a noninvasive NIRS-based parameter for autoregulation measurement, especially Cox, which represents the correlation coefficient between arterial blood pressure and regional saturation, rSO_2_, assuming that changes in tissue oxygen saturation are directly correlated with flow assuming a constant metabolic demand [21, 25]. Positive values of Cox suggest impaired autoregulation, whereas negative correlation indicate preservation of vasomotor response and preserved autoregulation [26].

Despite potentially useful, NIRS has a number of practical and methodological limitations [21], including the consistency of the assumed path length of the light as it passes through different tissues, and changes over time in hemoglobin concentration, SpO_2_, blood volume, and especially the risk of extracranial contamination. In general, despite the application of NIRS in non brain injured patients seems promising, these limitations have to be taken in account, and currently preclude the widespread and systematic application of this technique in practice [27, 28].

### Clinical applications

#### Neuromonitoring of cerebral oxygenation in the operating room

Intra and post-operative neurological complications are common even in non neurosurgical patients. The most common complications in these patients include delirium, post-operative cognitive decline, stroke, spinal cord ischemia, and can potentially increase mortality and morbidity [2, 29–31].

In particular, in some types of surgery such as vascular and cardiac surgery, the risk of major neurological complications is very high, with reported rate of stroke of 7% after carotid stenting and of 3.2% after endarterectomy [29–31].

Similarly, neurocognitive dysfunction, including postoperative delirium, occur in nearly 50% of cases after cardiac surgery, and stroke in up to 2%, while postoperative dysfunction occurs in up to 42% [30]. However, neurological complications can also occur following non-high-risk surgeries, such as shoulder surgery, mainly caused by beach chair positioning and hypotension [32].

NIRS has been recently proposed in the settings of cardiac surgery both in the preoperative and intraoperative periods, with the aim to detect patients at risk of neurological complications and allowing the identification and treatment of episodes of acute cerebral hypoperfusion [33–35]. In fact, NIRS can provide information on the changes of cerebral oxygenation before and during the perioperative period, raising the suspect of intraoperative cerebral events. During carotid surgery, a regional cerebral oxygenation of less than 50% seems to be an indicator of hypoperfusion; similarly, during aortic surgery, lumbar values of rSO_2_ of < 75% for 15 min can predict the occurrence of spinal cord injury [36]. Recently, a large systematic review and meta-analysis which assessed preoperative rSO_2_ values in cardiac surgery found mean baseline value of 66% and median reference range of rSO_2_ values between 51 and 82% [36].

According to available evidence, intraoperative intervention is required in case of a reduction of > 10% of the rSO_2_ value compared to baseline, or if it falls below the absolute value of 50%, as the sensitivity of NIRS in detecting cerebral ischemia ranges from 60 to 100%, with good specificity (94–98%).

In a study including 90 elderly patients undergoing orthopedic surgery, it was found that patients with cognitive dysfunction at 3 months after surgery had more frequently episodes of intraoperative episodes of cerebral desaturation and at least a 10% decrease from preoperative rSO_2_, suggesting a significant relationship between cerebral blood oxygen saturation detection and neurological complications in this population [37].

Importantly, studies are consistent in observing that not only the absolute single value, but the time spent below 50% of rSO_2_ is significantly associated with the occurrence of postoperative complications, such as delirium [36].

Therefore, NIRS should be used in the context of a multimodal neuromonitoring approach, and its values should be cautiously interpreted, considering the baseline values and its trend, as well as pre-operative patient’s status.

#### Emergency department and intensive care unit

Non invasive neuromonitoring in the Emergency Department (ED) and ICU may be a valuable complement to clinical diagnosis and radiological images in non-primarily brain-injured patients [38, 39].

Neurological complications are common in patients admitted to the ED and ICU especially those who are admitted for sepsis, metabolic, renal or hepatic insufficiency, intoxication and cardiac arrest [39, 40].

NIRS has been evaluated to assess cerebral perfusion and autoregulation after cardiac arrest and detect episodes of cerebral desaturation showing a correlation between its values and severity of illness and with variable association between rSO_2_ value and outcome [41, 42]. Similarly, NIRS has shown to be useful to assess episodes of cerebral desaturation in patients with acute distress respiratory syndrome and COVID-19 during respiratory manipulations and the use of respiratory rescue therapies [7, 43–46], as well as in septic patients where cerebral desaturations were found to be predictors of neurological sequelae [47, 48].

Sepsis-associated brain dysfunction (SABD) is considered as cerebral dysfunction following sepsis, in absence of direct or primarily structural central nervous system infection, it affects up to 70% of patients with sepsis admitted to the ICU and is associated with worse outcomes. Systemic inflammation leads to altered cerebral blood flow, disruption of brain blood barrier and altered autoregulation [49]. Recent evidence suggest that cerebral autoregulation is altered in half of the patients with sepsis and is associated with the development of SABD [49].

Similarly, pregnant women who develop pre-eclampsia have frequently impaired cerebral autoregulation [50]. In this context, NIRS has demonstrated to be able to detect cerebral oxygenation impairment in severe preeclamptic parturients, thus suggesting that disorders in cerebral microcirculation and/or changes in cerebral oxygenation may occur in this population [51].

Despite the diagnostic and prognostic potentiality of non-invasive multimodal neuromonitoring in the ED, the use of these techniques is still limited in these settings and are currently more frequently adopted in the post-emergency settings after ICU admission.

## Conclusions

**Fig. 2:**
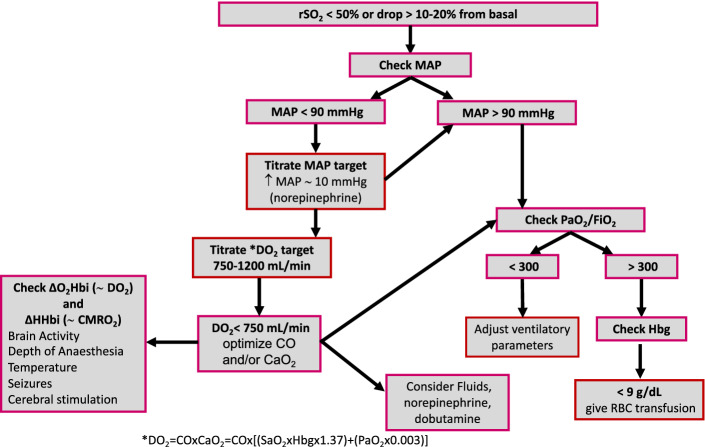
Algorithm for decision making in case of cerebral desaturation. *rSO*_*2*_ cerebral oxygen saturation, *MAP* mean arterial pressure, *PaO2* arterial partial pressure of oxygen, *FiO2* fraction of inspired oxygen, *Hbg* hemoglobine, *RBC* red blood cells, *DO*_2_ oxygen delivery, *CO* cardiac output, *CaO*_2_ arterial content of oxygen, *O*_2_*Hbi* oxygenated haemoglobin, *HHbi* deoxygenated haemoglobin, *CMRO*_2_ cerebral metabolic rate of oxygen

Increased evidence suggests that non invasive cerebral oximetry is a key monitoring strategy in the management of patients undergoing anaesthesia or sedation in the intensive care whose primary injury does not involved the brain. This seems to be relevant not only in the perioperative settings, but also in the emergency department and the ICU. NIRS has the advantage of being a non-invasive, low-cost, safe and a bedside available tool, with a great potential for diagnosis and treatment of patients at risk of neurological complications. In Fig. 2, we propose a decisional algorithm for the management of patients who develop episodes of cerebral desaturation. Further studies and guidelines are warranted in order to confirm the findings in present literature, and training and teaching programs are urgently needed to implement the use of this neuromonitoring tool in daily clinical practice.
